# Matrix Metalloproteinase-20 Over-Expression Is Detrimental to Enamel Development: A *Mus musculus* Model

**DOI:** 10.1371/journal.pone.0086774

**Published:** 2014-01-23

**Authors:** Masashi Shin, Yuanyuan Hu, Coralee E. Tye, Xiaomu Guan, Craig C. Deagle, Jerry V. Antone, Charles E. Smith, James P. Simmer, John D. Bartlett

**Affiliations:** 1 Department of Mineralized Tissue Biology and Harvard School of Dental Medicine, The Forsyth Institute, Cambridge Massachusetts, United States of America; 2 Department of Biologic and Materials Sciences, University of Michigan School of Dentistry, Ann Arbor, Michigan, United States of America; 3 Program in Endodontics, Harvard School of Dental Medicine, Boston Massachusetts, United States of America; 4 Facility for Electron Microscopy Research, Department of Anatomy & Cell Biology, and Faculty of Dentistry, McGill University, Montreal, QC, Canada; Casey Eye Institute, United States of America

## Abstract

**Background:**

Matrix metalloproteinase-20 (*Mmp20*) ablated mice have enamel that is thin and soft with an abnormal rod pattern that abrades from the underlying dentin. We asked if introduction of transgenes expressing *Mmp20* would revert this *Mmp20* null phenotype back to normal. Unexpectedly, for transgenes expressing medium or high levels of *Mmp20,* we found opposite enamel phenotypes depending on the genetic background (*Mmp20^−/−^* or *Mmp20^+/+^*) in which the transgenes were expressed.

**Methodology/Principal Findings:**

*Amelx-*promoter-*Mmp20* transgenic founder mouse lines were assessed for transgene expression and those expressing low, medium or high levels of *Mmp20* were selected for breeding into the *Mmp20* null background. Regardless of expression level, each transgene brought the null enamel back to full thickness. However, the high and medium expressing *Mmp20* transgenes in the *Mmp20* null background had significantly harder more mineralized enamel than did the low transgene expresser. Strikingly, when the high and medium expressing *Mmp20* transgenes were present in the wild-type background, the enamel was significantly less well mineralized than normal. Protein gel analysis of enamel matrix proteins from the high and medium expressing transgenes present in the wild-type background demonstrated that greater than normal amounts of cleavage products and smaller quantities of higher molecular weight proteins were present within their enamel matrices.

**Conclusions/Significance:**

*Mmp20* expression levels must be within a specific range for normal enamel development to occur. Creation of a normally thick enamel layer may occur over a wider range of *Mmp20* expression levels, but acquisition of normal enamel hardness has a narrower range. Since over-expression of *Mmp20* results in decreased enamel hardness, this suggests that a balance exists between cleaved and full-length enamel matrix proteins that are essential for formation of a properly hardened enamel layer. It also suggests that few feedback controls are present in the enamel matrix to prevent excessive MMP20 activity.

## Introduction

Dental enamel is the hardest tissue of the body, but it does not start that way. Enamel development (amelogenesis) can be defined as consisting of three stages; the secretory, transition, and maturation stages [1]. During the secretory stage the ameloblasts adjacent to the forming enamel elongate and secrete large quantities of protein into the enamel matrix. Approximately 90% of this protein is amelogenin [2]. Amelogenin has only one post-translational modification whereby Ser16 is phosphorylated [3], [4], but its transcripts undergo extensive alternative splicing [5], [6], [7] to generate as many as 16 X-chromosomal murine amelogenin mRNAs [8], [9], [10]. The secretory stage is when thin crystallite ribbons begin growing in length [11], [12], [13] and they stop elongating once ameloblasts have defined the full thickness of the enamel layer. Secretory stage enamel is very soft and has a cheese-like consistency. Once the ameloblasts progress to the transition stage, they shorten and greatly reduce protein secretion. During the subsequent maturation stage, the short columnar ameloblasts reabsorb the proteins they had previously secreted. This is when the enamel ribbons grow to their greatest amount in width and thickness [1]. Enamel consists of mineralized rods that are sometimes entwined (gnarled) in human molars [14] or may form groups of rods that pass across each other (decussating) as is observed in rodent incisors [15]. Each rod is formed by one ameloblast and contains approximately 10,000–40,000 crystallite ribbons [11]. Therefore, defective thin (hypoplastic) enamel suggests a developmental deficiency during the secretory stage when the crystallite ribbons are lengthening to establish the full thickness of the enamel layer. Erupted teeth with enamel that has reached full thickness, but is soft and not well mineralized (hypomaturation) suggests a developmental deficiency during the maturation stage when the crystallite ribbons grow in width and thickness and interlock. Fundamental developmental deficiencies may also affect both stages of enamel development (hypocalcified) and these cases typically have the most severely dysplastic enamel phenotypes [16].

Matrix metalloproteinase-20 (MMP20; enamelysin) is a tooth specific MMP [17] that is secreted into the enamel matrix during the secretory and transition stages of enamel development [18], [19], [20], [21]. DNA encoding the *Mmp20* catalytic domain was previously deleted from the mouse genome to identify the role of MMP20 in enamel development [22]. *Mmp20* null mice did not process amelogenin properly, had altered enamel rod patterns and had hypoplastic enamel that broke away from dentin. A subsequent study showed that relative to wild-type controls, *Mmp20* null mice had an overall enamel mineral content that was reduced by 50% and an enamel hardness that was decreased by 37% [23]. Although *Mmp20* is only expressed from the secretory through transition stages, it was concluded that protein processing by MMP20 is necessary so that the ameloblasts can efficiently remove proteins during the maturation stage. Kallikrein-related peptidase-4 (KLK4) is a serine proteinase secreted during the maturation stage to further cleave enamel matrix proteins prior to their export out of the hardening enamel [24]. However, KLK4 can apparently not compensate for a lack of prior MMP20 activity so the *Mmp20* null mouse enamel retains matrix proteins and is therefore softer than normal. Seven different human *MMP20* mutations are known to cause autosomal recessive non-syndromic *amelogenesis imperfecta* [25], [26], [27], [28], [29], [30].

Here we sought to determine if introduction of transgenes that express low, medium or high levels of MMP20 in the *Mmp20* null background would revert the murine *Mmp20* enamel phenotype back to normal. The high and medium expressing transgenes did result in a marked improvement in enamel development over that of the *Mmp20* null mice. Unexpectedly, the high or medium expressing transgene caused malformed enamel when present in the wild-type background suggesting that a critical balance exists between cleaved and full-length enamel matrix proteins that are essential for proper hardening of the enamel layer. Beyond a specific threshold, too much MMP20 activity disrupts this balance and high quantities of cleavage products disrupt normal enamel development.

## Materials and Methods

### Ethics Statement

All animals used in this study were housed in an Association for Assessment and Accreditation of Laboratory Animal Care (AAALAC) accredited facilities (animal welfare assurance number: A3051-01) and were treated humanely based on a protocol 11-021 approved by the Institutional Animal Care and Use Committee (IACUC) at The Forsyth Institute. Experimental protocols were designed along institutional and National Institutes of Health guidelines for the humane use of animals.

### Construction of the *Mmp20* Transgene

C57BL/6 mice were sacrificed on post-natal day 5, and first molars were removed. Total RNA was extracted with Trizol (Invitrogen, Carlsbad, CA, USA) and converted into cDNA with SuperScript II reverse transcriptase (Invitrogen). *Mmp20* cDNA was amplified for ligation into the pCR2.1-TOPO vector (Invitrogen). Primers used for this amplification were: forward, 5′-TGG CGC GCC AGA GGA GAT GAA GGT GCT ACC TGC C-3′ and reverse, 5′-AGC GAT CGC CAC TGC AGG TGC TAC CAG GAA GTA GG-3′. These primers amplified 2278 bp of the *Mmp20* cDNA that included the start codon and 821 bp of 3′ non-coding region. The beginning of the forward primer contained an *AscI* restriction enzyme site and the beginning of the reverse primer contained a *SgfI* restriction enzyme site to enable the PCR product’s directional ligation into the vector. This vector was previously constructed to contain 4639 bp of mouse *AmelX* promoter region including the first exon (non-coding) and first intron. Immediately 3′ to the *Mmp20* cDNA insertion site was 1127 bp of *AmelX* 3′ non-coding region that included amelogenin polyadenylation signals [31].

### Generation of *Mmp20* Transgenic Mice and Breeding with *Mmp20^−/−^* Mice

The *Amelx* promoter*-Mmp20* cDNA with the 3′-*Amelx* non-coding region was excised from the vector by restriction digestion with *NotI*-*SrfI,* purified with a Qiaquick gel extraction kit (Qiagen, Germantown, MD, USA) and microinjected into fertilized oocytes for surgical transfer to recipients. Germline transmission was determined by PCR analyses of genomic DNA obtained from tail biopsies. PCR primers used to identify the presence of the transgene were: 5′-GAA AAT GGT TTG CAG CAT CA-3′, and 5′-CTT GCC ACC ATC TCG CCA GCC-3′. For mouse genotyping, genomic DNA was isolated from tails and the Extract-N-Amp Kit (Sigma) was used for PCR reactions. PCR primer sequences for determining *Mmp20* genotypes were: 5′-CTG CGT CCC CAG ACT TTT GAT TT-3′, and 5′-GCT TTT CAT GGC CAG AAT GCT CT-3′, to detect the ablated allele and 5′-AAG TAG ACT GAA GTC AGG AGA GCC-3′, and 5′-CTG TAG TGG TGA CCC TAG TCA TCT T-3′, to detect the wild-type allele. Offspring carrying the *Mmp20* transgene (Tg) were mated with *Mmp20* ablated mice [22] to produce Tg positive *Mmp20^+/−^* and these mice were bred to generate both Tg positive *Mmp20^−/−^* and Tg positive Mmp20^+/+^ mice. Therefore, each transgenic founder mouse line had a similar genetic background.

### Quantitative Real-time PCR (qPCR)

RNA extracted from incisor enamel organ of adult mice or first molar enamel organs of 5 day-old mice were used to determine relative expression levels of *Mmp20* as a function of a stably expressed internal reference control gene (18S rRNA) as previously described [32], [33]. Primers were: *Mmp20* forward 5′-GCC TCT TCC CAG GTG AAC CCA -3′), *Mmp20* reverse (5′-ACG CAT GCA GGG CCA TCT GT-3′); 18S forward (5′-GTA ACC CGT TGA ACC CCA TT-3′), 18S reverse 5′-CCA TCC AAT CGG TAG TAG CG-3′. Reactions were performed on a Roche LightCycler 480 using the following program: 3 min at 95°C for initial denaturation, and 95°C 15 sec, 58°C 15 sec, 72°C 15 sec for 40 cycles, followed by a melting curve. Each time point was obtained by triplicate qPCR analysis, and all expression levels are presented as relative ratios to the wild-type incisor or day 5 first molar data.

### Protein Gels, Immunoblotting and Zymography

Mandibular incisors were extracted from adult mice and first molars were removed from 5-day old mouse pups. The mineral was rapidly dissolved by submerging the teeth in 2 mL of 0.17 M HCl/0.98% formic acid for 2 h at 4°C. Undissolved material was removed by centrifugation at 3,500 g for 5 min at 4°C. The samples were then dialyzed against water overnight and lyophilized for immunoblot or zymography analysis. The lyophilized proteins were weighed and eluted into sample buffer. For immunoblots, an equal amount of protein was loaded in each lane and was run on SDS-PAGE and transferred to a membrane. Immunoblots were performed with antiserum specific for the N-terminus of active MMP20 (Abcam, Cambridge, MA, USA; Ab39037, 1∶2500 in TBST) or with antiserum for amelogenin (rabbit anti-recombinant antibody [34], 1∶3000 in TBST). Casein zymogram gels were purchased from Invitrogen and electrophoresis was performed at a constant voltage of 120 V for 1 h. After electrophoresis, gels were washed twice for 30 min in 2.5% TritonX-100. Gels were incubated for 2 days at 37°C in 50 mM Tris-HCl buffer (pH 7.2) containing 10 mM CaCl_2_ and were then stained with Coomassie Brilliant Blue (CBB) R-250 solution (0.1% CBB R-250, 10% acetic acid, and 50% methanol) for 15 min and destained with 30% methanol and 10% acetic acid until clear bands of substrate lysis were observed. Silver staining was performed according to the manufacture’s protocol (Amersham Biosciences, NJ, USA).

### Photographs of Mouse Teeth

Soft tissues were removed from right half-mandibles of adult mice. Photographs of the incisors and lingual views of molars were taken using a Nikon SMZ745T microscope and Leica DFC400 digital camera at 30x magnification.

### MicroComputed Tomography (µCT)

Adult mouse incisor enamel was assessed for mineralization levels by µCT. Hemi-mandibles with soft tissues removed were immersed in saline and scanned in a µCT-40 (Scanco Medical, Wayne PA, USA) with the following settings: 70 kV, 114 mA, and 0.01 mm isotropic voxels. Images were processed with µCT-40 evaluation software and ImageJ was used to orient the incisors so that enamel layer mineralization could be clearly observed.

### Scanning Electron Microscopy (SEM) of Mandibular Incisors

Adult mouse mandibular incisors with soft tissue removed were fixed in 5% glutaraldehyde overnight with rotation, washed in PBS x 3 and were embedded in Epon. Perpendicular cuts through incisors were made 8 mm from their apical ends. Incisors were then re-embedded in Chastolite AC (Eager polymers, Chicago IL, USA) with the cutting plane face down and were allowed to harden at room temperature overnight. The surfaces were then ground with silicon carbide (400, 800 & 1200 grit) prior to polishing with a 1 µm diamond polish paste. The surfaces were sputter-coated with gold for conventional SEM or were carbon coated for backscattering SEM. SEM was performed as described previously [31]. Five-level pseudo-color mapping was performed as described previously [35] using ImageJ (http://rsb.info.nih.gov/ij/) on TIFF images that were normalized to have the same mean gray-level intensities for mineralized dentin (mature dentin mineral shows no significant changes with or without MMP20 expression). For pseudo-colorization of images, gray levels 1–57 were assigned as black, 58–97 as white, 98–162 as blue, 163–192 as orange, and 193–255 as red. These settings were saved in a look-up table (LUT) and applied to selected SEM images.

### Vickers Microhardness Testing

Erupted portions of mandibular incisors from wild-type, *Mmp20^−^* ^/*−*^ and *Mmp20* transgenic mice were washed and dehydrated with graded alcohol and acetone. Incisors were embedded sagittally in hard-formulation epoxy embedding medium (EpoFix, EMS, Hatfield, PA, USA). Samples were ground and polished to 0.25 mm with diamond suspensions (EMS). The polished samples were tested for enamel microhardness on an M 400 HI testing machine (Leco, St. Joseph, MI, USA). Testing was performed with a load of 25 g for 5 sec with a Vickers tip. Twenty indentations *per* sample were performed on at least 4 teeth *per* group and averaged.

### Statistics

Unpaired t-tests were performed using GraphPad Prism version 5.0 (GraphPad Software, San Diego, CA) to analyze the significance of differences in enamel thickness and enamel hardness. T-tests were also performed for analyzing the significance of qPCR results.

## Results

### Production of Transgenic Mice

**Figure 1:**
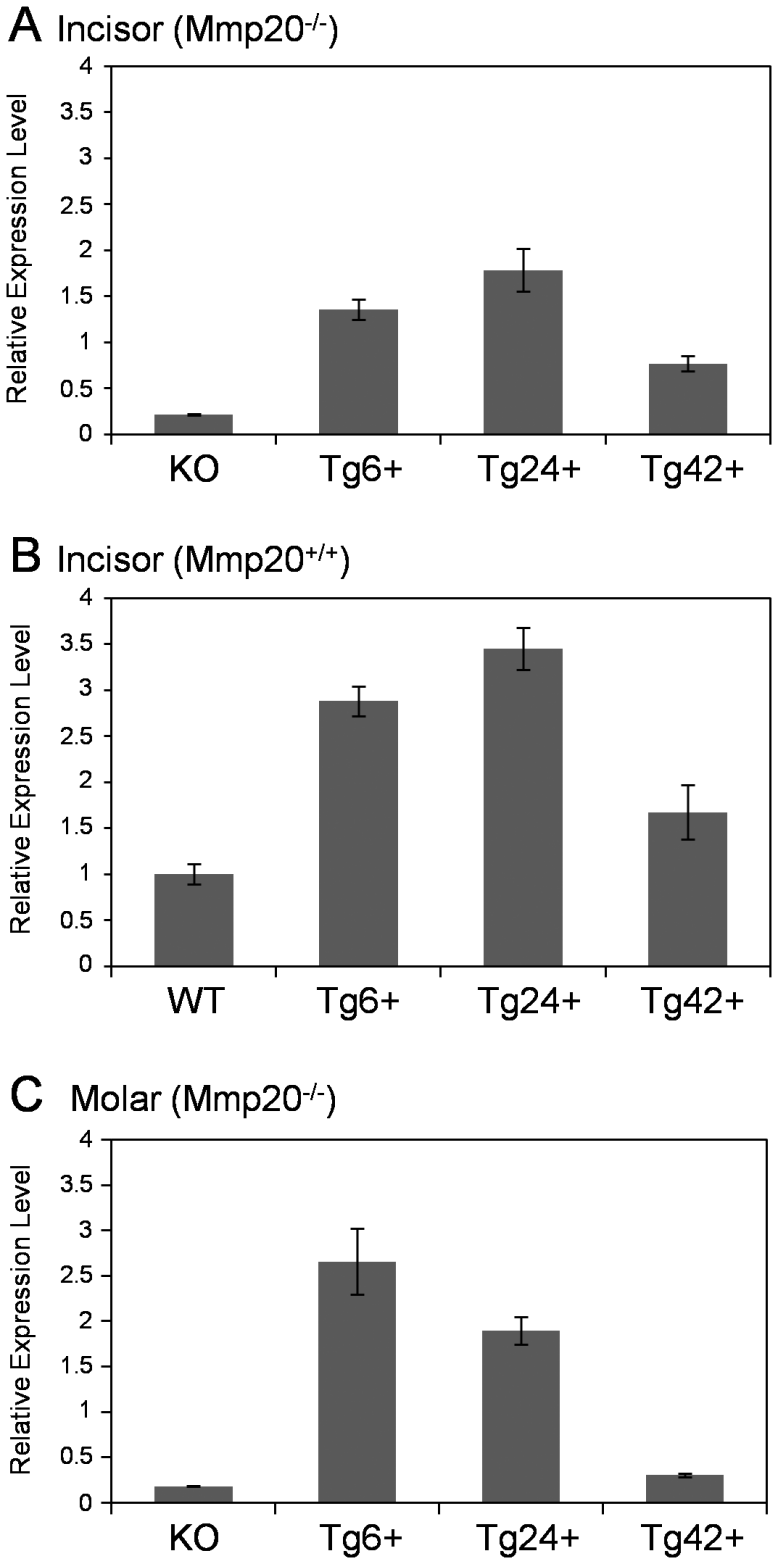
Relative *Mmp20* transcript expression levels in murine enamel organs from offspring of three different transgenic founder mice and controls. qPCR was performed to quantify *Mmp20* expression. Shown are *Mmp20* expression results from enamel organs of adult incisors (A, B) that continuously erupt and that therefore contain all stages of enamel development. Also shown are *Mmp20* expression levels in 5 day-old first molar enamel organs (C) that are predominantly in the secretory stage of enamel development. Incisors from mice transgenic for the Tg6 or Tg24 transgenes each expressed significantly more *Mmp20* (P<0.05) whereas the Tg42 transgenic mice expressed significantly less *Mmp20* (P<0.05) compared to endogenous levels when the transgenes were present in the *Mmp20* null background (A). As expected, total expression of *Mmp20* was highest when the transgenes were present in the *Mmp20* wild-type background where combined expression of endogenous and transgenic *Mmp20* was quantified (B). In contrast to incisors, transgene expression in the molars was highest in the Tg6 mice with Tg24 expressing mid-levels and, like the incisors, Tg42 was expressed at the lowest levels in molars (C). KO, *Mmp20* knockout; WT, wild-type.

A transgene was designed such that 4639 bp of the mouse amelogenin promoter was inserted 5’ to the mouse *Mmp20* cDNA and 1127 bp of amelogenin 3′ non-coding region that included amelogenin polyadenylation sites was inserted immediately 3′ to the *Mmp20* cDNA. The construct was sequenced in its entirety to confirm its integrity. Three transgenic founder mice were selected based on the ability of their incisor enamel organs to express low (Tg42), intermediate (Tg6) or high (Tg24) levels of *Mmp20* transgene transcripts in the *Mmp20* null background (Fig. 1A). We also performed qPCR on incisor enamel organs to confirm that the total level of wild-type and transgene *Mmp20* transcripts were increased when the transgenes were present in the wild-type background (Fig. 1B). Interestingly, transgene expression in first molar enamel organs from 5 day-old mice that are predominately in the secretory stage of enamel development, displayed expression levels that were different from incisors. In molars, Tg6 transgenic mice had the highest level of expression followed by the Tg24 transgene and the Tg42 transgene was expressed in mice at lower than wild-type endogenous levels (Fig. 1C). Note that minimal *Mmp20* null (KO) amplification products may have been generated because the qPCR primers located upstream of the deleted region (exon 5) encoding the *Mmp20* catalytic domain.

**Figure 2:**
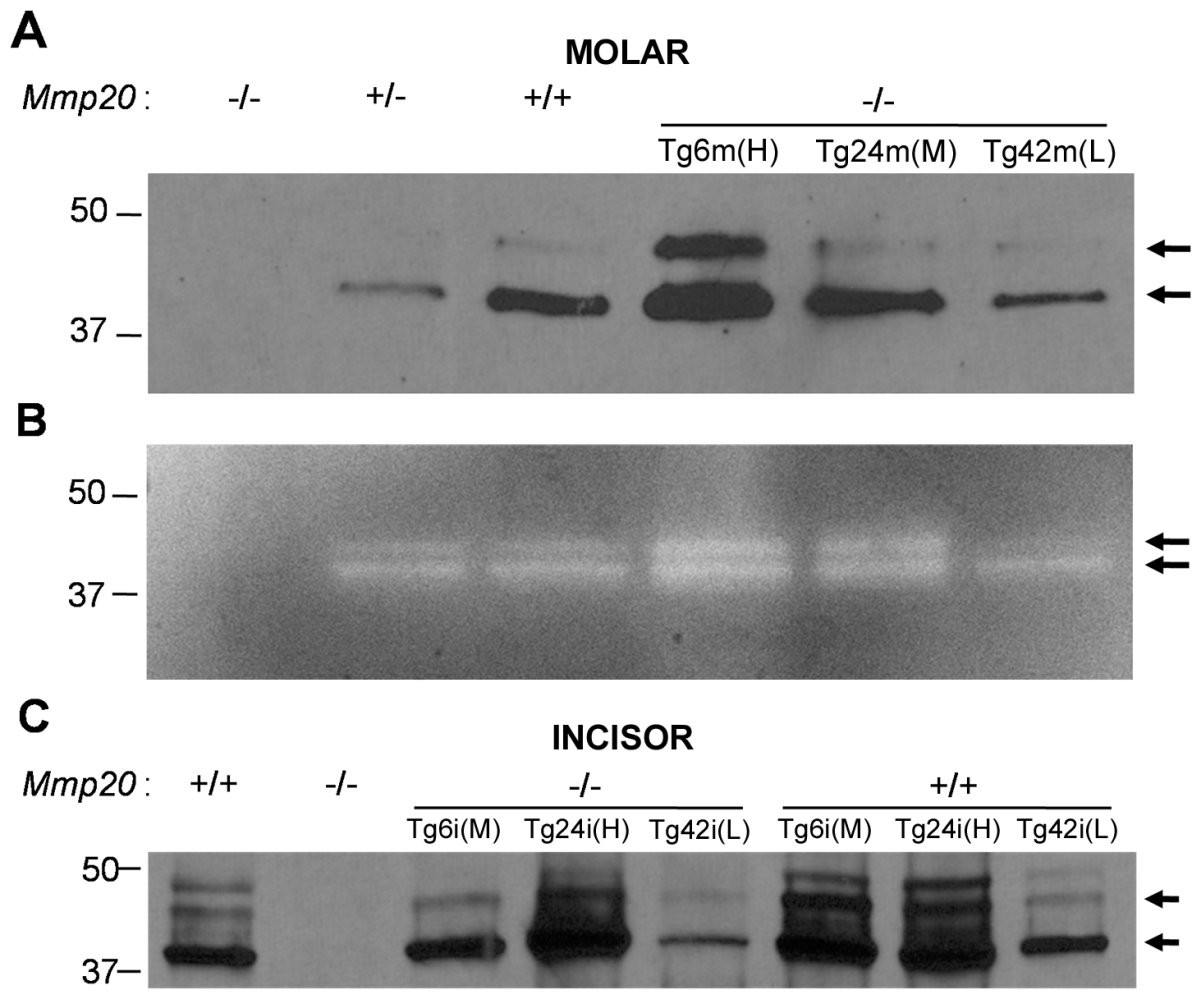
Assessment of MMP20 protein content from extracted enamel. Immunoblots performed on proteins extracted from 5 day-old molars (A) or adult incisors (C) assessed MMP20 quantity. Zymography of extracted enamel from 5 day-old molars assessed MMP20 proteolytic activity (B). MMP20 was not detected in *Mmp20* null (–/–) mouse enamel, low levels were observed in the heterozygous (+/–) enamel and wild-type (+/+) enamel had more MMP20 protein than did the heterozygotes. In molar (m) enamel from *Mmp20* null mice, the Tg6 transgene [Tg6m (H)] expressed the highest quantities of MMP20 followed by the Tg24m (M) transgene at mid-levels with the Tg42m (L) transgene expressing the lowest levels of MMP20. The Tg42m (L) transgene expressed lower MMP20 amounts than were present in enamel from wild-type mice (A). Zymography results for MMP20 activity (B) were consistent with the immunoblot results. In contrast to the molar results, immunoblots performed on extracted incisor (i) enamel showed that enamel from Tg24i (H) transgenic mice contained the highest amount of MMP20 protein followed by Tg6i (M) enamel and then Tg42i (L) transgenic enamel which, like the molars, also contained less MMP20 than wild-type enamel (C). As expected, enamel from transgenes expressed in the *Mmp20* wild-type background had total MMP20 quantities that were higher for each transgene than were observed in the null background. Arrows point to the MMP20 doublet bands that each represents an active form of MMP20.

To confirm that transcript expression paralleled MMP20 protein levels, we performed immunoblots on proteins extracted from molars or incisors and performed zymography on 5 day-old first molar enamel matrix proteins. Consistent with the qPCR results for 5 day-old molars (m), the Tg6 transgenic mice expressed the most MMP20 protein (H) whereas Tg42 transgenic mice expressed the least (L). The wild-type (+/+) mice expressed more MMP20 protein than the *Mmp20* heterozygous (+/–) mice while the null (–/–) mice expressed no MMP20 (Fig. 2A). The zymography results supported the immunoblot results by demonstrating that more active MMP20 was present in enamel from Tg6m (H) transgenic 5 day-old mouse first molars than was present in the Tg24m (M) (medium expression) or Tg42m (L) (lowest expression) transgenic mouse molars (Fig. 2B). Conversely, in extracted incisor (i) enamel the Tg24i (H) transgene was expressed at the highest level followed by the Tg6i (M) and Tg42i (L) transgenes respectively. When the transgenes were expressed in wild-type mice that express endogenous MMP20, the quantities of MMP20 extracted from incisor enamel from each of the transgenic mice was increased over the levels observed when each transgene was present in the *Mmp20* null background (Fig. 2C). Therefore, the order of transgene expression level in mouse incisors was Tg24i (H)>Tg6i (M)>>Tg42i (L) and was Tg6m (H)>Tg24m (M)>>Tg42m (L) for molars. The highest levels of total MMP20 expression (endogenous and transgenic) were observed in wild-type mice expressing the Tg24 transgene in incisors or the Tg6 transgene in molars.

### Examination of Transgenic Mouse Teeth

**Figure 3:**
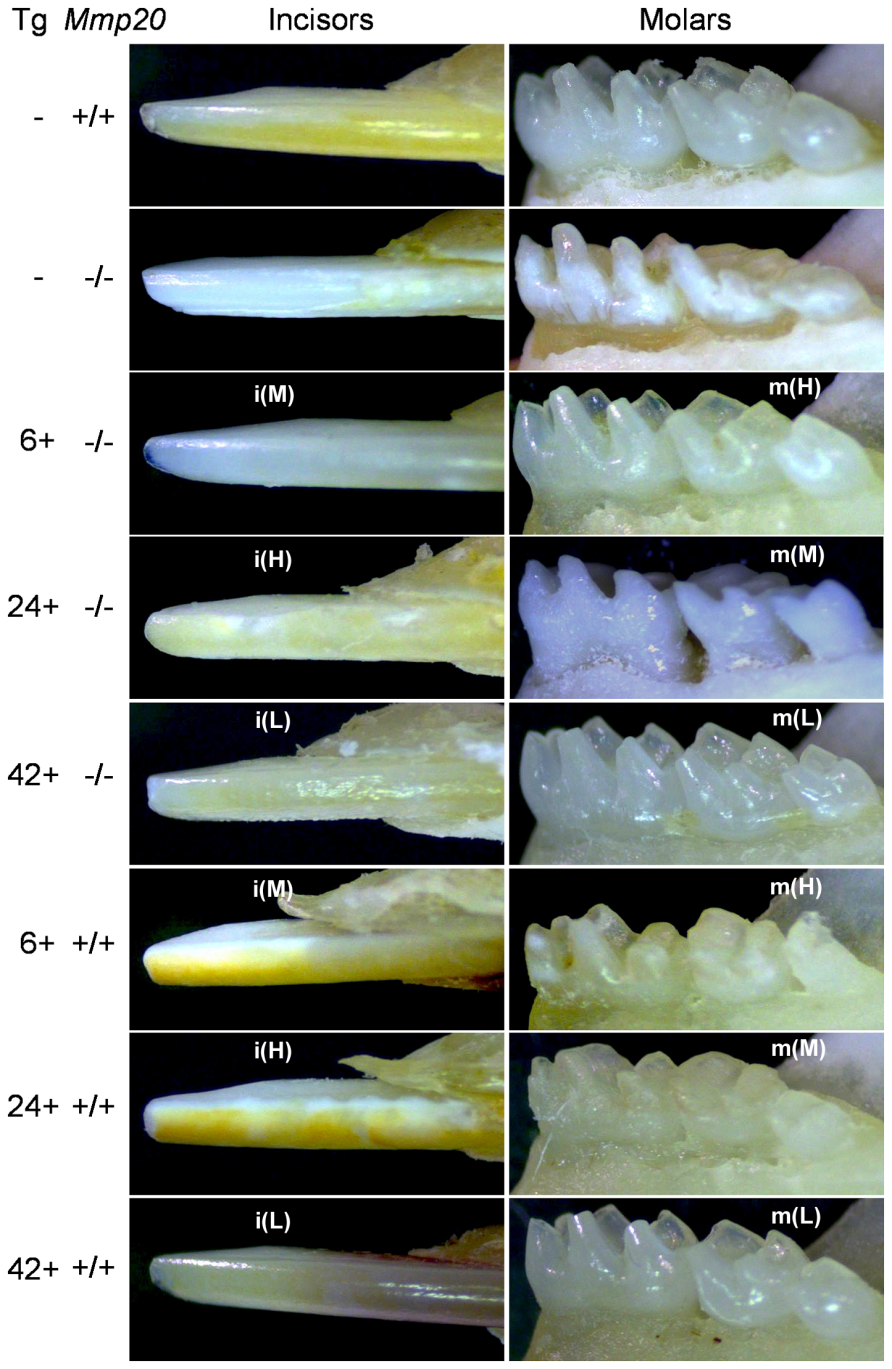
Assessment of control and *Mmp20* transgenic enamel by light microscopy. Wild-type control (Tg– *Mmp20^+/+^*) incisors had a sharp incisal tip and the characteristic yellow-brown coloration. The molars contained a fully thick enamel layer and the cusp tips were well defined. In contrast, *Mmp20* null (Tg– *Mmp20^−/−^*) incisors had a blunted tip and showed little or no enamel and no yellow-brown color. The molars were worn and the remaining cusp tips appeared thin from abrasion and absence of a full thickness enamel layer. For the transgenes in the *Mmp20* null background (Tg+ *Mmp20^−/−^*): The Tg6 transgenic enamel had a sharp incisal tip, but the yellow-brown color was mostly missing while the molars appeared fully recovered from the null phenotype. The Tg24 transgenic animals had incisors that appeared sharp and somewhat yellow-brown in color, but the enamel surface was rough and appeared slightly chalky rather than translucent. The molar cusp tips were worn and the enamel layer appeared hypoplastic. The Tg42 transgenic mice had incisors that were blunted with a rough enamel surface that was mildly yellow-brown color with a chalky appearance like the Tg24 transgenic incisors. The molars appeared well formed and fully recovered from the null phenotype. For the transgenes in the *Mmp20* wild-type background (Tg+ *Mmp20^+/+^*): The Tg6 transgenic mice had well pigmented but blunted incisors with a chalky-white appearance and rough enamel surface. The molars were severely compromised having abraded cusp tips with pitted enamel surfaces. The Tg24 transgenic enamel appeared almost identical to the Tg6 enamel in the wild-type background. In contrast, Tg42 transgenic incisors had a sharp tip and smooth enamel, but with almost no yellow-brown color. The Tg42 transgenic molars appeared no different than the wild-type molars. The observed Tg6 transgenic enamel was the best example of an overall recovery (both incisors and molars) from the *Mmp20* null phenotype.

Mandibular incisors and molars from *Mmp20* transgenic mice were examined under a light microscope (Fig. 3). Each transgene was present in the wild-type or *Mmp20* null background. The wild-type incisor (Tg– *Mmp20* ^+/+^) had the characteristic yellow-brown color and had a sharp tip. The wild-type molars had distinctive cusp tips that were thick with a full thickness enamel layer. In contrast, *Mmp20* null incisors (Tg– *Mmp20^−^* ^/*−*^) showed no yellow-brown color, had enamel that peeled away from the underlying dentin and had a blunted tip. *Mmp20* null molars had worn cusp tips or had thin cusp tips because the enamel had abraded away. As expected, expression of the transgenes in the *Mmp20* null background did recover some (Tg24, Tg42 molars) or nearly all (Tg6, Tg24 incisors) of the otherwise dysplastic enamel phenotype. In the wild-type background, although the teeth from the lowest expressing transgene (Tg42) appeared normal, the teeth from the two highest expressing transgenes (Tg6, Tg24) unexpectedly had blunted incisor tips and had molars with worn cusp tips. The enamel phenotype in the presence of these two transgenes was much worse in the wild-type background when compared to the enamel phenotype when these transgenes were expressed in the *Mmp20* null background.

### Incisor µCT Analysis

**Figure 4:**
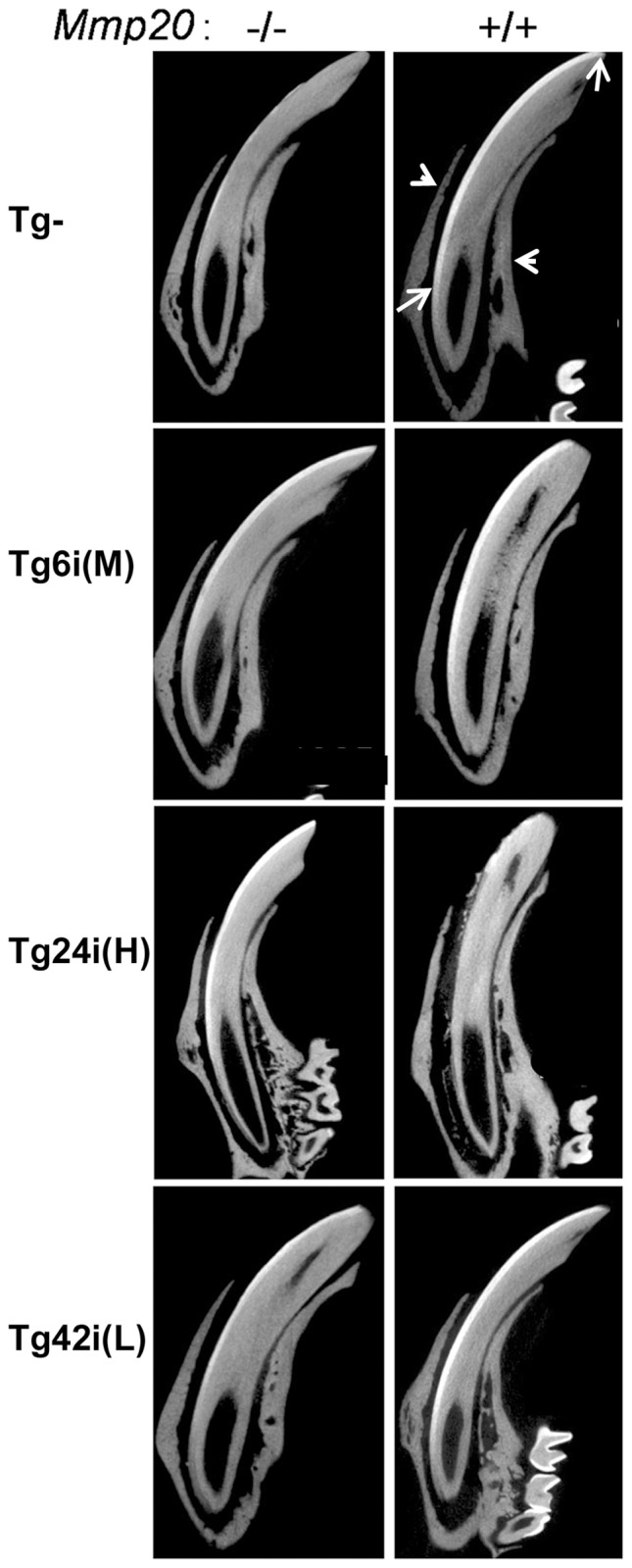
µCT analyses of incisor enamel from *Mmp20* transgenics and controls. Enamel on rodent incisors is present only on the labial side (arrows). The presented longitudinally oriented incisors were reconstructed from µCT images. The incisors protrude from bone (arrowheads) and are arranged so that the labial side is to the left. The wild-type incisors (Tg– MMP20^+/+^, top right panel) have a bright line of mineralized enamel that extends from the apical region (bottom arrow) to the labial incisal tip (top arrow). This mineralized enamel was mostly missing from the *Mmp20* null incisors (Tg– *Mmp20^−/−^*, top left panel). The transgenic incisors in the null background (Tg+ *Mmp20^−/−^*) recovered some or most of the enamel layer along the labial surface. When the transgenes were present in the wild-type background (Tg+ Mmp20^+/+^), the enamel layer seemed relatively normal on incisors from mice transgenic for Tg6i (M) or Tg42i (L), but it was severely disrupted on the Tg24i (H) *Mmp20^+/+^* incisors. For incisors, Tg24 was the highest, Tg6 the middle and Tg42 the lowest expressing transgene.

Rodent incisors have enamel on only their labial sides. Presented in the micrographs of Figure 4 are incisors with their labial sides oriented to the left hand side of the incisor. On the labial side of the wild-type incisors (Tg– *Mmp20* ^+/+^, top right panel) a translucent line (layer) of enamel is present that begins at the apical end (bottom arrow) of the incisor encased in bone (arrowheads) and extends up to the incisal tip (top arrow). This layer of enamel is very thin, spotty or absent along the labial edge of the transgene negative *Mmp20* null (Tg– *Mmp20^−^* ^/*−*^, top left panel) mouse incisors. An enamel layer was present on each transgene positive incisor in the *Mmp20* null background and in the wild-type background an enamel layer was present on incisors of mice expressing the Tg6i (M) or Tg42i (L) transgenes. However, wild-type mice with the highly expressing Tg24i (H) transgene showed very little enamel on their incisors. These µCT data confirm the light microscope results demonstrating that high levels of MMP20 over-expression in normal mice results in dysplastic enamel.

### Backscattered SEM

**Figure 5:**
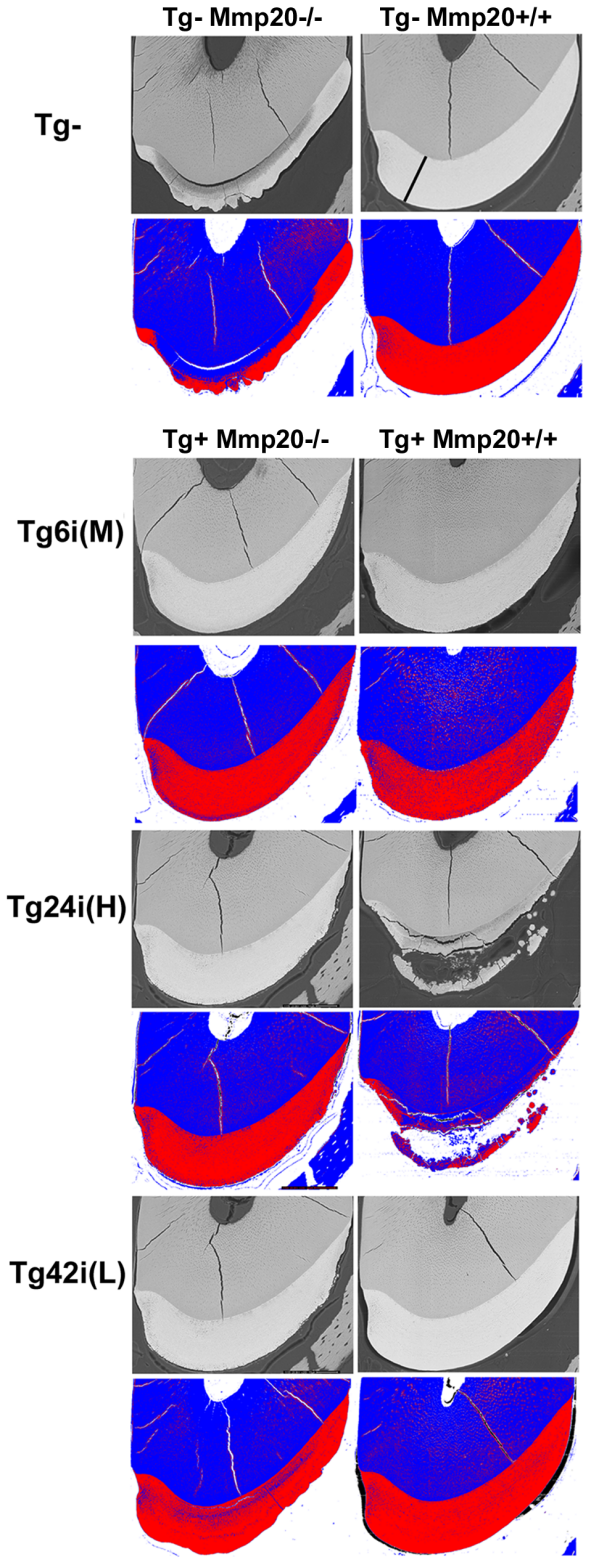
Assessment of incisor enamel mineralization by backscatter SEM and pseudo-color mapping. Backscatter images of incisor cross sections were sectioned at a site located 8-color mapping of the image above. Color mapping allows easier visualization of differences in enamel mineralization within the enamel layer. Blue and white colors indicate decreased mineralization relative to the highly mineralized red color. For wild-type mice (Tg– Mmp20+/+), color mapping shows that enamel on the mesial (left) side is not quite as mineralized as is the rest of the enamel layer at this level of sectioning just prior to where the incisor erupts into the mouth. The *Mmp20* null mouse (Tg– Mmp20–/–) enamel illustrates the variations in appearance of the enamel layer that is typical for these teeth. For transgenes in the *Mmp20* null background, Tg6i (M) transgenic incisors had less mineralized enamel especially at the lateral (right) side and the surface. Tg24i (H) transgenic incisors showed similar results as well as an irregular enamel surface. Enamel from mice transgenic for Tg42i (L) in the *Mmp20* null background had rougher surfaces and large areas of poor mineralization near the dentin-enamel junction. For the transgenes present in the *Mmp20* wild-type background (Tg+ Mmp20+/+), the situation was reversed from what occurred in the *Mmp20* null background. Mice transgenic for Tg6i (M) had poorly mineralized enamel throughout while Tg24i (H) transgenic mice had disorganized and clearly disrupted enamel formation. Conversely, the Tg42i (L) transgene in the wild-type background appeared relatively normal. In the top right panel, the black line extending from the dentin enamel junction to the outer edge of the enamel shows where enamel thickness measurements were obtained.

The more highly mineralized the tissue, the more calcium there is to scatter electrons. Therefore, to characterize the degree of enamel mineralization for each of the transgenes in the wild-type and *Mmp20* null backgrounds, we performed backscattered SEM imaging and used computer software to assign different colors to images that fell within one of five ranges of grey level intensity [35]. In Figure 5, the top panel is the backscattered image and the panel beneath it is the same image that has been pseudo-colorized for assessment of mineralization levels. The most highly mineralized tissues appear red and less mineralized areas are in blue color.

The first column of Figure 5 shows incisor cross sections from *Mmp20^−/−^* mice and the second column shows incisor cross sections from *Mmp20^+/+^* mice. For wild-type mice (Tg– Mmp20+/+), the colorized sections demonstrate that enamel is more highly mineralized than dentin. As was previously observed in *Mmp20^−/−^ mice* (Tg– Mmp20–/–) [35], the outer enamel layer is more mineralized than the inner enamel layer and the characteristic nodules present on *Mmp20* null enamel can be observed protruding from the outer enamel layer. The Tg+ *Mmp20^−^* ^/*−*^ column shows that Tg6i (M) transgenic mice increased the degree of enamel mineralization to almost normal levels. However, the lateral sides of the enamel layer appeared slightly softer than normal. The Tg24i (H) transgenic mice also reverted the *Mmp20* null enamel phenotype almost completely back to normal. Although mineralization near the dentin-enamel junction (DEJ) was slightly less than normal and the enamel surface on the lateral side was not completely smooth. In contrast, Tg42i (L) transgenic mice expressed the least amount of MMP20 and had enamel that was not fully mineralized with a prominent line of softer enamel near the DEJ. This incisor also had a rough enamel surface.

The degree of enamel mineralization in wild-type mice expressing various transgenes was striking because it was reversed from what occurred in the *Mmp20* null background. The low transgene expression level in Tg42i (L) transgenic mice resulted in perfectly normal enamel whereas the enamel from Tg6i (M) or Tg24i (H) transgenic mice in the wild-type background was significantly compromised. Incisor enamel from Tg6i (M) transgenic mice in the wild-type background appeared softer than normal especially at the lateral edges. The outer edge of the Tg24i (H) transgenic mouse enamel appeared to have broken away from the remaining soft enamel that in many places looked no more well mineralized than the underlying dentin. Clearly *Mmp20* over-expression compromised the degree of enamel mineralization.

### Quantification of Enamel Thickness

**Figure 6:**
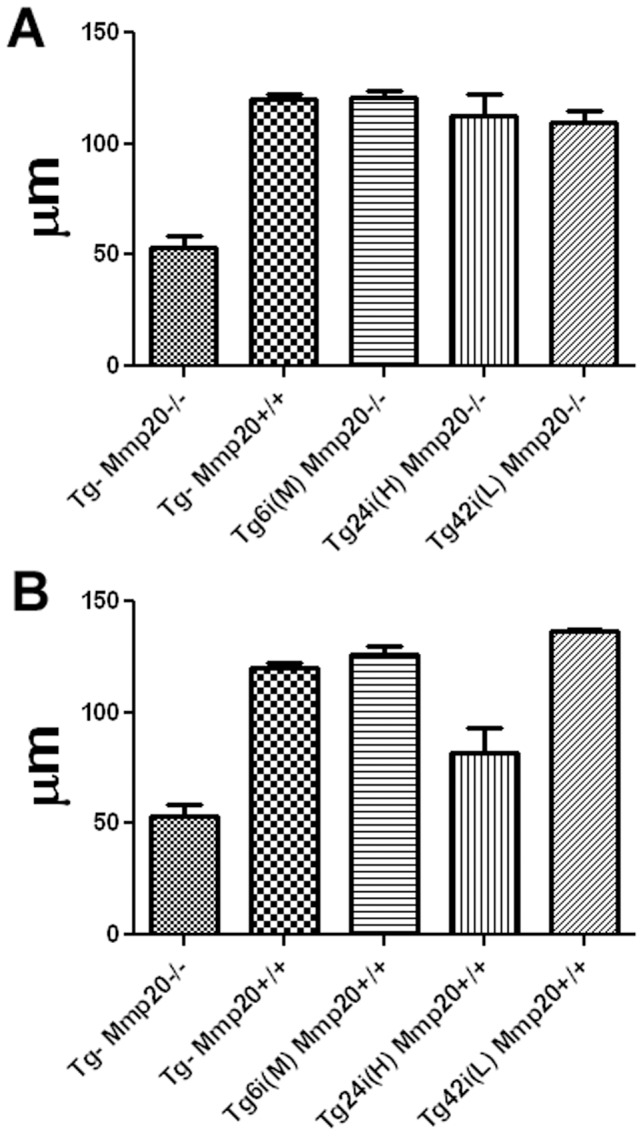
Assessment of enamel thickness in incisor cross-sections. Thickness was measured in the widest portion of the enamel layer of each incisor cross section as illustrated by the line in the top right panel of Figure 5. Each bar (genotype) on this graph represents enamel measurements from three different mouse incisors. No significant difference in enamel thickness was observed among wild-type and transgenes present in the *Mmp20* null background (A). Each of the three transgenes brought the null enamel back to its normal thickness. When the transgenes were present in the wild-type background (B), the most highly expressed transgene [Tg24i (H)] had an enamel layer that was significantly thinner (P<0.001) than wild-type enamel.

All backscatter SEM incisor cross section images were cut at a location near where they erupt from the labial alveolar bone crest (8 mm from the apical end of the tooth). Care was taken to insure a perpendicular cut through the incisor to prevent an artificial increase in enamel thickness due to oblique angled cuts. Thickness was measured in the thickest portion of the enamel at the centro-mesial side of each incisor cross section. Each bar in Figure 6 represents the measurement of an incisor cross section from three different mice. Each transgene expressed in the *Mmp20* null background reverted the *Mmp20* null enamel back to normal thickness. No significant differences in enamel thickness existed among the wild-type and *Mmp20* null transgene positive incisors (Fig. 6A). Therefore, minimal quantities of MMP20 allow the enamel to attain its full thickness. With the exception of transgene Tg24i (H), transgene expression in the wild-type background resulted in enamel that was of normal thickness. In the incisor enamel organ, the Tg24i (H) transgene expresses the highest levels of *Mmp20* and this combined with the endogenously expressed *Mmp20* resulted in a thinner than normal enamel layer (P<0.001) (Fig. 6B). Therefore, low levels of *Mmp20* expression are sufficient to achieve normally thick enamel, but high levels interfere with the developmental processes necessary to achieve a fully thick enamel layer.

### Quantification of Enamel Hardness

**Figure 7:**
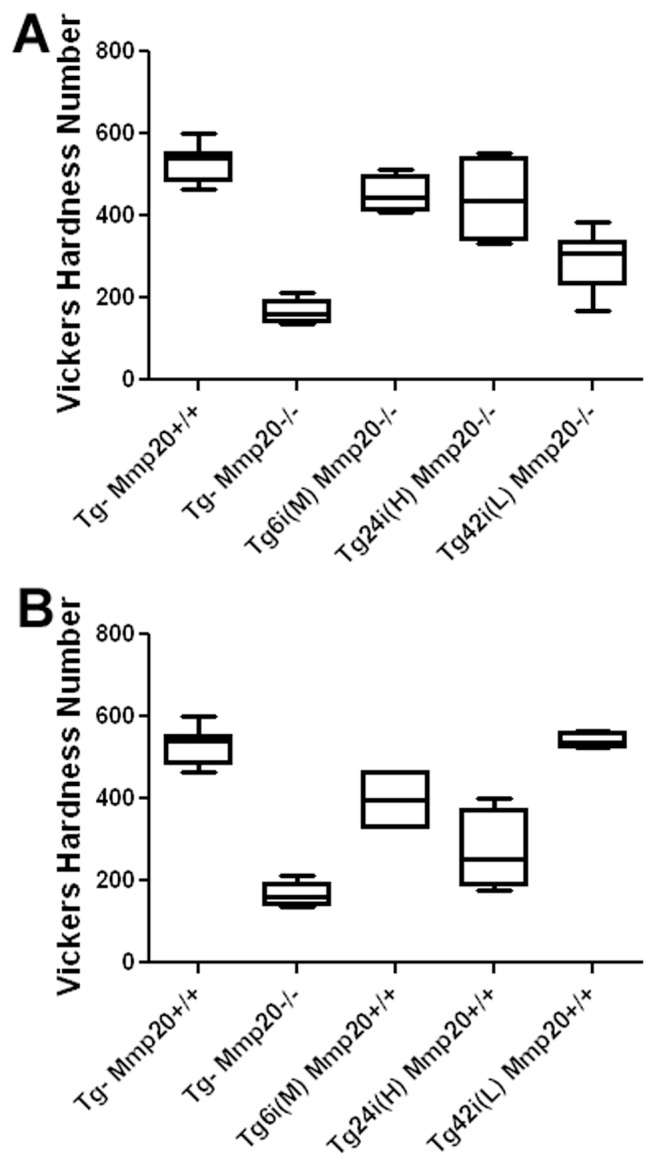
Assessment of incisor microhardness on incisor longitudinal sections. Approximately 20 indentations throughout the enamel layer were obtained per incisor and the results were average to generate one data point for the graph. Measurements from at least 4 incisors from each genotype were used to generate a bar on the graph. Whiskers denote the data range and the horizontal line within the box represents the median microhardness value. When present in the *Mmp20* null background (A), the Tg6i (M) *Mmp20^−/−^* (N = 4) enamel was slightly softer (P<0.05) than wild-type mouse enamel (Tg– *Mmp20^+/+^*, N = 8), but the Tg24i (H) *Mmp20^−/−^* (N = 4) enamel had a wider range of microhardness values and was therefore not significantly different from wild-type. The Tg42i (L) *Mmp20^−/−^* (N = 7) enamel was much softer than enamel from wild-type mice and this difference was highly significant (P<0.0001). In contrast, each of the transgenic mice had enamel that was harder than the enamel from the *Mmp20* null mouse incisors (Tg– *Mmp20^−/−^*, N = 11) and these differences were all highly significant (P<0.0001). Enamel hardness values positively correlated to the level of transgene expression in mouse incisors when the transgenes were in the *Mmp20* null background. When present in the *Mmp20* wild-type background (B), the Tg6i (M) *Mmp20^+/+^* (N = 4) incisor enamel was softer (P<0.01) than enamel from wild-type mice (Tg– *Mmp20^+/+^*, N = 8) and the Tg24i (H) *Mmp20^+/+^* (N = 4) enamel was much softer than enamel from wild-type mice (P<0.0001). However, no difference in enamel hardness was observed between wild-type and Tg42i (L) *Mmp20^+/+^* (N = 4) enamel. Enamel hardness values negatively correlated to the level of transgene expression in mouse incisors when the transgenes were in the wild-type background.

Incisors were embedded in epoxy resin and were ground and polished in preparation for microhardness testing. Each bar represents hardness measurements for incisors from at least 4 different mice. The Tg– *Mmp20* ^+/+^ and the Tg– *Mmp20^−^* ^/*−*^ samples were each pooled separately so these bars represent hardness measurements for incisors from at least 8 different mice. Vickers hardness values plus or minus the standard error of the mean for wild-type (Tg– *Mmp20* ^+/+^) and *Mmp20* null (Tg– *Mmp20^−^* ^/*−*^) were 530.2±15.54 N = 8 and 164.9±8.063 N = 11 respectively. Enamel hardness was not significantly different between incisors from wild-type and Tg24i (H) *Mmp20^−^* ^/*−*^ mice (440.1±52.77 N = 4). However, Vickers harness values demonstrated that wild-type enamel (Fig. 7A) was significantly harder than Tg6i (M) *Mmp20^−^* ^/*−*^ enamel (451.9±21.93 N = 4; P<0.05) and was also significantly harder than Tg42i (L) *Mmp20^−^* ^/*−*^ enamel (291.3±26.48 N = 7; P<0.0001). Therefore, each transgene in the *Mmp20* null background increased the level of enamel hardness over that of the null mouse incisors, but only the highly expressed Tg24i (H) transgenic mice had enamel hardness values comparable to wild-type controls.

Microhardness results for transgenes expressed in *Mmp20* ^+/+^ background were strikingly opposite to the results observed in the *Mmp20^−^* ^/*−*^ background. The only commonality between the groups was that transgene expression resulted in harder enamel than was present on the *Mmp20* null (Tg– Mmp–/–) incisors (Fig. 7B). In the wild-type background the Tg42i (L) transgenic mice had Vickers hardness values (540.2±9.192 N = 4) that were not significantly different from wild-type enamel. However, wild-type enamel was significantly harder than Tg6i (M) *Mmp20* ^+/+^ enamel (395.7±39.45 N = 4; P<0.01) and was also significantly harder than Tg24i (H) *Mmp20* ^+/+^ enamel (271.1±48.71 N = 4; P<0.0001). Therefore MMP20 must be expressed within a tight range for the development of enamel with optimal hardness.

### Enamel Matrix Protein and Amelogenin Banding Patterns

**Figure 8:**
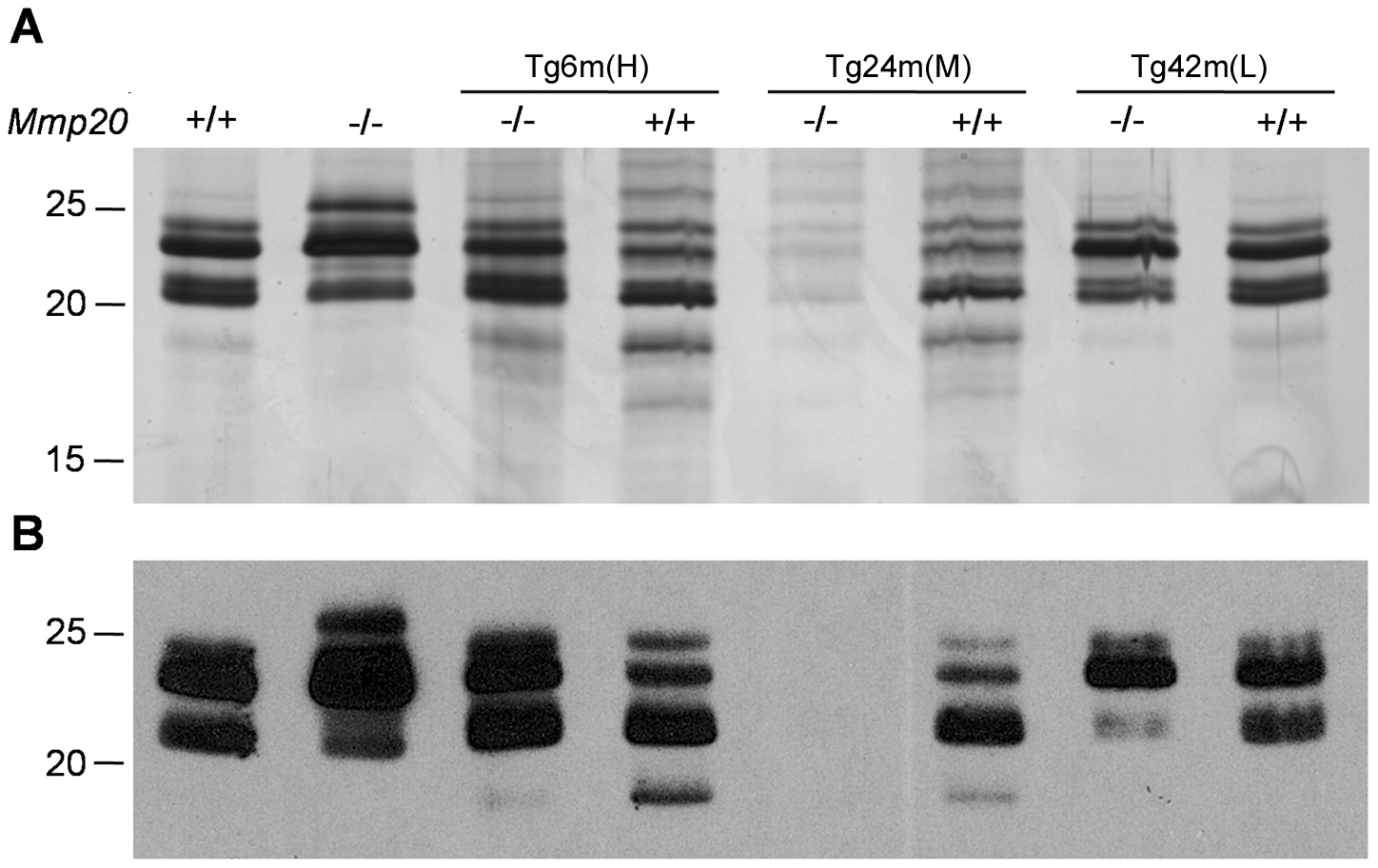
Enamel matrix protein profile and immunoblotting of amelogenin splice and cleavage products. Total extracted enamel matrix proteins (mostly amelogenins) from 5-day old molars were run on an SDS PAGE gel (A). Comparison of the first two lanes containing extracted wild-type or *Mmp20* null enamel matrix proteins demonstrate that the null enamel has a prominent band at approximately 27 kDa that is only weakly present in the wild-type lane indicating that this band is cleaved by MMP20. Also a doublet just above 20 kDa was present in the wild-type lane but was a single band in the null lane indicating that one of the wild-type bands is an MMP20 cleavage product. The protein profile for the Tg6m (H) transgene in the *Mmp20^−/−^* background was similar to that of the protein extracted from wild-type enamel. However, the protein profile for Tg6m (H) and Tg24m (M) in the *Mmp20^+/+^* background had a strong band below the 20 kDa marker and a weaker band beneath that indicating that more cleavage products than normal were present. Both transgenes in the wild-type background had bands above 20 kDa that were less prominent than the bands observed from wild-type enamel with no transgene. In contrast, regardless of the *Mmp20* background, the Tg42m (L) positive mice had protein banding patterns that looked substantially like the wild-type results. Amelogenin immunoblot results from the various geneotypes (B) were similar to the protein gel results. An approximate 27-kDa amelogenin band was present in the *Mmp20* null but not wild-type lanes. In the wild-type background, both Tg6m (H) and Tg24m (M) transgenes had prominent amelogenin bands that located below 20 kDa and the bands above 20 kDa appeared less prominent than those observed in the Tg– wild-type lane. Also the amelogenin profile of Tg42m (L) in the wild-type background looked similar to that of wild-type mice.

To determine if enamel matrix proteins are processed differently from normal when high levels of MMP20 are present, we performed an enamel matrix total protein analysis and performed immunoblotting for amelogenin. Five day-old first molars were collected and proteins were extracted from their developing enamel. The enamel proteins were run on an SDS-PAGE gel (Fig. 8A) and were subjected to immunoblotting for amelogenin (Fig. 8B). As was previously observed [22], the *Mmp20* null enamel has a prominent amelogenin band of approximately 27 kDa that was weakly present in enamel from wild-type mice indicating that the 27-kDa band is cleaved by MMP20. A band below 20 kDa was present in the wild-type enamel, but not in the *Mmp20* null mouse enamel suggesting that it was an MMP20 cleavage product. This lower band was present in greatest quantities in the Tg6m (H) *Mmp20* ^+/+^ and Tg24m (M) *Mmp20* ^+/+^ mouse enamel and the higher molecular weight bands were less abundant for these two transgenes. In contrast, the low level expression of the Tg42m (L) transgene precluded the detection of the band below 20 kDa in the null background and the higher molecular weight bands were present at approximately wild-type levels in the Tg42m (L) *Mmp20* ^+/+^ mouse enamel. Apparently the band below 20 kDa is an MMP20 cleavage product that accumulates in greater quantities at the expense of the higher molecular weight bands when more MMP20 is present.

## Discussion

*Mmp20* transgenes driven by the amelogenin promoter and amelogenin downstream non-coding sequence were introduced into *Mmp20* ablated mice to determine if the severe *Mmp20* null phenotype could be reversed back to normal. Low, medium and high expressing transgenic mouse lines were selected to assess the level of MMP20 activity necessary to revert the null phenotype and for their effect on enamel when present in the wild-type background. These experiments were performed on enamel from adult mouse incisors and on enamel from 5 day-old mouse pup first molars. Since rodent incisors continuously erupt, every stage of enamel development is present along the erupting incisor. Although the secretory stage of enamel development is only approximately 2 mm long near the basal end of the mouse incisor [36], extraction of total incisor enamel contained detectable MMP20 on immunoblots (Fig. 2). However, enamel matrix protein profiles from total incisor protein contain both MMP20 and KLK4 cleavage products. Therefore to assess total protein or amelogenin MMP20 cleavage profiles (Fig. 8), we used 5 day-old mouse pup first molars that were predominantly in the secretory stage of development when MMP20 is the only proteinase present in the enamel matrix.

It was surprising to find that transgene expression levels differed depending on whether expression was assessed in incisors or molars. Perhaps the localization of a given transgene within the mouse genome will favor expression in the continuously erupting incisor or favor expression in developing pup molars. Interestingly, the immunoblots for MMP20 were different depending on if the molar or incisor was probed. The wild-type molars (Fig. 2A, lane 3) show two MMP20 bands as indicated by the arrows while the wild-type incisors (Fig. 2C. lane 1) show three bands. Both bands identified by the arrows are catalytically active [37]. Therefore, the higher molecular weight MMP20 band observed from extracted incisor enamel may be the inactive zymogen. The results for Tg24m (M) transgene expression in molars were difficult to interpret. The qPCR, immunoblot and zymography results suggest that mice transgenic for Tg24 expressed this transgene at levels higher than the endogenous wild-type levels. However, the enamel present on molars in the *Mmp20^−/−^* background appeared malformed (Fig. 3) and although we tried several times, we had difficulty extracting total enamel proteins from Tg24m (M) *Mmp20^−/−^* mouse molars (Fig. 8). It appeared that amelogenin in these molars was not prevalent. Perhaps enamel formation was delayed in these mice so that their molars had a thin enamel layer with little protein. Paradoxically, amelogenins were successfully extracted from Tg24m (M) *Mmp20^+/+^* molar enamel suggesting that transgenic MMP20 may lack an essential function that the endogenous MMP20 provides.

The most surprising finding was that for incisor enamel, the mid-level expressing transgene Tg6i (M) and the high-level expressing transgeneTg24i (H) almost completely recovered the *Mmp20* null enamel phenotype. But, when these same transgenes were present in the wild-type background, the incisor enamel was severely compromised and enamel from mice transgenic for high-level expressing Tg24i (H) was more severely compromised than mice expressing the mid-level Tg6i (M) transgene (Fig. 5). Therefore, the endogenously expressed MMP20 combined with transgenic MMP20 activity resulted in poor quality enamel. This has striking implications. The results suggest that enamel matrix proteins have primary and secondary MMP20 cleavage sites and imply that if the secondary sites are cleaved too early due to much higher than normal MMP20 activity, malformed enamel will result.

In support of this interpretation, previous studies have shown that MMP20 rapidly cleaves amelogenin near its C-terminus and that progressive amelogenin degradation occurs with longer MMP20 incubation times [38]. A more detailed study demonstrated that MMP20 processes amelogenins into groups of cleavage products that accumulate and that are only slowly degraded further by MMP20 [39]. Ameloblastin [40] and enamelin [41], [42] are the two other non-proteinase components of the enamel matrix. Although, these proteins are present in the matrix in far less abundance compared to amelogenin, they are nonetheless essential for murine enamel development [43], [44]. Enamelin is so quickly hydrolyzed by MMP20 [45] that apparently all of its cleavage sites are primary MMP20 sites. Ameloblastin, however, is cleaved early by MMP20 at its N-terminus and secondary cleavages occur near the C-terminus [46], [47]. Furthermore, during the secretory stage the enamel crystallites predominantly grow in length. However, over time MMP20 degrades enamel proteins and allows some growth of crystals in width and thickness, particularly in the deeper enamel [48]. MMP20 is the only known secretory stage enamel matrix proteinase. So, prolonged incubation with MMP20 may allow this growth. Again, this suggests that MMP20 has secondary enamel matrix protein cleavage sites that permit growth in width and thickness. Therefore, it is likely that a delicate balance exists in the enamel matrix among full-length proteins, proteins that are cleaved quickly, and proteins that are cleaved slowly and that too much MMP20 activity disrupts this balance.

Although transgene expression levels in the *Mmp20* null background correlated positively with enamel quality, no difference among transgenic and wild-type mice existed with regard to incisor enamel thickness. With the exception of Tg24i (H) transgenic mice, the same was true for the transgenes expressed in the wild-type background. Therefore, for all but the most highly expressing transgene in the wild-type background, the enamel crystallites were able to extend to their full length to form a normally thick enamel layer. This suggests that enamel matrix protein cleavage products are more important for achieving fully mineralized enamel than they are for achieving a fully thick enamel layer. This fits well with current theories stating that full-length enamel proteins are responsible for crystallite elongation and that their cleavage products are more important for providing a supporting structure for the lengthening crystallites [19], [49]. Perhaps very low *Mmp20* expression as observed in the Tg42i (L) *Mmp20^−/−^* enamel leaves behind un-cleaved proteins that interfere with proper mineralization and perhaps high *Mmp20* expression levels cause early cleavage of secondary sites that compromise the crystallite support structure. When *Mmp20* is expressed at very high levels such as in Tg24i (H) *Mmp20^+/+^* incisor enamel, the full-length enamel matrix proteins may be cleaved so quickly that they cannot adequately support continued lengthening of the crystallites. In any case, achievement of a normally thick enamel layer may occur over a wide range of *Mmp20* expression levels, but achievement of normal enamel hardness has a much narrower range.

A prior study demonstrated that *Mmp20* heterozygous mice expressed half the quantity of *Mmp20* transcripts and their enamel contained approximately half the quantity of MMP20 protein as did wild-type mice [50]. This same study showed that no difference existed in enamel hardness between wild-type and heterozygous mice. Therefore, half the normal level of MMP20 activity does not significantly affect enamel mineralization. But from the present transgene results, it appears that if the level of MMP20 activity falls much below 50%, enamel mineralization will be adversely affected.

We conclude that enamel matrix proteins must be cleaved by MMP20 at a rate that is permissible for optimal enamel development. If the rate of cleavage is too fast or too slow, the enamel will be defective. Also, although the chances for under expression of MMP20 are much greater in humans than for its over expression, the finding that too high a level of MMP20 activity leads to enhanced degradation of enamel matrix proteins implies that there are likely few inhibitors or feedback controls over this proteinase once it is secreted.

## References

[1] Hu JC, Chun YH, Al Hazzazzi T, Simmer JP (2007) Enamel formation and amelogenesis imperfecta. Cells Tissues Organs 186: 78–85. 17627121 10.1159/000102683

[2] Termine JD, Belcourt AB, Christner PJ, Conn KM, Nylen MU (1980) Properties of dissociatively extracted fetal tooth matrix proteins. I. Principal molecular species in developing bovine enamel. J Bio Chem 255: 9760–9768. 7430099

[3] Fincham AG, Moradian-Oldak J (1995) Recent advances in amelogenin biochemistry. Connect Tiss Res 32: 119–124. 10.3109/03008209509013713 7554907

[4] Takagi T, Suzuki M, Baba T, Minegishi K, Sasaki S (1984) Complete amino acid sequence of amelogenin in developing bovine enamel. Biochemical and Biophysical Research Communications 121: 592–597. 6732825 10.1016/0006-291x(84)90223-7

[5] Gibson CW, Golub E, Ding WD, Shimokawa H, Young M, et al (1991) Identification of the leucine-rich amelogenin peptide (LRAP) as the translation product of an alternatively spliced transcript. Biochemical and Biophysical Research Communications 174: 1306–1312. 1996994 10.1016/0006-291x(91)91564-s

[6] Lau EC, Simmer JP, Bringas P Jr, Hsu DD, Hu CC, et al (1992) Alternative splicing of the mouse amelogenin primary RNA transcript contributes to amelogenin heterogeneity. Biochemical and Biophysical Research Communications 188: 1253–1260. 1445358 10.1016/0006-291x(92)91366-x

[7] Salido EC, Yen PH, Koprivnikar K, Yu LC, Shapiro LJ (1992) The human enamel protein gene amelogenin is expressed from both the X and the Y chromosomes. American Journal of Human Genetics 50: 303–316. 1734713 PMC1682460

[8] Li Y, Yuan ZA, Aragon MA, Kulkarni AB, Gibson CW (2006) Comparison of body weight and gene expression in amelogenin null and wild-type mice. Eur J Oral Sci 114 Suppl 1190–193. 16674684 10.1111/j.1600-0722.2006.00286.x

[9] Bartlett JD, Ball RL, Kawai T, Tye CE, Tsuchiya M, et al (2006) Origin, splicing, and expression of rodent amelogenin exon 8. J Dent Res 85: 894–899. 16998127 10.1177/154405910608501004 PMC2229627

[10] Baba O, Takahashi N, Terashima T, Li W, DenBesten PK, et al (2002) Expression of alternatively spliced RNA transcripts of amelogenin gene exons 8 and 9 and its end products in the rat incisor. The Journal of Histochemistry and Cytochemistry: official journal of the Histochemistry Society 50: 1229–1236. 12185201 10.1177/002215540205000910

[11] Daculsi G, Menanteau J, Kerebel LM, Mitre D (1984) Length and shape of enamel crystals. Calcif Tissue Intl 36: 550–555. 10.1007/BF02405364 6441627

[12] Cuisinier FJ, Steuer P, Senger B, Voegel JC, Frank RM (1992) Human amelogenesis. I: High resolution electron microscopy study of ribbon-like crystals. Calcif Tissue Int 51: 259–268. 1422970 10.1007/BF00334485

[13] Kerebel B, Clergeau-Guerithault S, Brion M (1975) Ultrastructural odontological study of a case of Papillon-Lefevre disease. Ann Anat Pathol (Paris) 20: 283–292. 174463

[14] Boyde A (1989) Enamel. In: Oksche A, Vollrath L, editors. Handbook of Microscopic Anatomy. Berlin: Springer-Verlag. pp. 309–473.

[15] Reith EJ, Ross MH (1973) Morphological evidence for the presence of contractile elements in secretory ameloblasts of the rat. Arch Oral Biol 18: 445–448. 4515973 10.1016/0003-9969(73)90170-2

[16] Becerik S, Cogulu D, Emingil G, Han T, Hart PS, et al (2009) Exclusion of candidate genes in seven Turkish families with autosomal recessive amelogenesis imperfecta. Am J Med GenetA 149A: 1392–1398. 10.1002/ajmg.a.32885 PMC4264544 19530186

[17] Turk BE, Lee DH, Yamakoshi Y, Klingenhoff A, Reichenberger E, et al (2006) MMP-20 Is Predominately a Tooth-Specific Enzyme with a Deep Catalytic Pocket that Hydrolyzes Type V Collagen. Biochemistry 45: 3863–3874. 16548514 10.1021/bi052252o PMC2536712

[18] Bartlett JD, Ryu OH, Xue J, Simmer JP, Margolis HC (1998) Enamelysin mRNA displays a developmentally defined pattern of expression and encodes a protein which degrades amelogenin. ConnectTissue Res 39: 101–109. 10.3109/03008209809023916 11062992

[19] Bartlett JD, Simmer JP (1999) Proteinases in developing dental enamel. Crit Rev Oral Biol Med 10: 425–441. 10634581 10.1177/10454411990100040101

[20] Fukae M, Tanabe T, Uchida T, Lee SK, Ryu OH, et al (1998) Enamelysin (matrix metalloproteinase-20): localization in the developing tooth and effects of pH and calcium on amelogenin hydrolysis. J Dent Res 77: 1580–1588. 9719031 10.1177/00220345980770080501

[21] Begue-Kirn C, Krebsbach PH, Bartlett JD, Butler WT (1998) Dentin sialoprotein, dentin phosphoprotein, enamelysin and ameloblastin: tooth-specific molecules that are distinctively expressed during murine dental differentiation. Eur J Oral Sci 106: 963–970. 9786327 10.1046/j.0909-8836.1998.eos106510.x

[22] Caterina JJ, Skobe Z, Shi J, Ding Y, Simmer JP, et al (2002) Enamelysin (matrix metalloproteinase 20)-deficient mice display an amelogenesis imperfecta phenotype. J Biol Chem 277: 49598–49604. 12393861 10.1074/jbc.M209100200

[23] Bartlett JD, Beniash E, Lee DH, Smith CE (2004) Decreased mineral content in MMP-20 null mouse enamel is prominent during the maturation stage. J Dent Res 83: 909–913. 15557396 10.1177/154405910408301204

[24] Simmer JP, Hu Y, Lertlam R, Yamakoshi Y, Hu JC (2009) Hypomaturation Enamel Defects in Klk4 Knockout/LacZ Knockin Mice. J Biol Chem 284: 19110–19121. 19578120 10.1074/jbc.M109.013623 PMC2707199

[25] Kim JW, Simmer JP, Hart TC, Hart PS, Ramaswami MD, et al (2005) MMP-20 mutation in autosomal recessive pigmented hypomaturation amelogenesis imperfecta. J Med Genet 42: 271–275. 15744043 10.1136/jmg.2004.024505 PMC1736010

[26] Ozdemir D, Hart PS, Ryu OH, Choi SJ, Ozdemir-Karatas M, et al (2005) MMP20 active-site mutation in hypomaturation amelogenesis imperfecta. J Dent Res 84: 1031–1035. 16246936 10.1177/154405910508401112 PMC1850238

[27] Papagerakis P, Lin HK, Lee KY, Hu Y, Simmer JP, et al (2008) Premature stop codon in MMP20 causing amelogenesis imperfecta. J Dent Res 87: 56–59. 18096894 10.1177/154405910808700109 PMC2692082

[28] Lee SK, Seymen F, Kang HY, Lee KE, Gencay K, et al (2010) MMP20 hemopexin domain mutation in amelogenesis imperfecta. J Dent Res 89: 46–50. 19966041 10.1177/0022034509352844 PMC3318044

[29] Gasse B, Karayigit E, Mathieu E, Jung S, Garret A, et al.. (2013) Homozygous and Compound Heterozygous MMP20 Mutations in Amelogenesis Imperfecta. J Dent Res. 10.1177/0022034513488393 23625376

[30] Wang SK, Hu Y, Simmer JP, Seymen F, Estrella NM, et al (2013) Novel KLK4 and MMP20 mutations discovered by whole-exome sequencing. J Dental Res 92: 266–271. 10.1177/0022034513475626 PMC3576998 23355523

[31] Chun YH, Lu Y, Hu Y, Krebsbach PH, Yamada Y, et al (2010) Transgenic rescue of enamel phenotype in Ambn null mice. J Dent Res 89: 1414–1420. 20940352 10.1177/0022034510379223 PMC3085845

[32] Kubota K, Lee DH, Tsuchiya M, Young CS, Everett ET, et al (2005) Fluoride induces endoplasmic reticulum stress in ameloblasts responsible for dental enamel formation. J Biol Chem 280: 23194–23202. 15849362 10.1074/jbc.M503288200 PMC12931973

[33] Pfaffl MW (2001) A new mathematical model for relative quantification in real-time RT-PCR. Nucleic Acids Res 29: e45. 11328886 10.1093/nar/29.9.e45 PMC55695

[34] Simmer JP, Lau EC, Hu CC, Aoba T, Lacey M, et al (1994) Isolation and characterization of a mouse amelogenin expressed in Escherichia coli. Calcif Tissue Int 54: 312–319. 8062146 10.1007/BF00295956

[35] Hu Y, Hu JC, Smith CE, Bartlett JD, Simmer JP (2011) Kallikrein-related peptidase 4, matrix metalloproteinase 20, and the maturation of murine and porcine enamel. Europ J Oral Sci 119 Suppl 1217–225. 10.1111/j.1600-0722.2011.00859.x PMC3281808 22243249

[36] Smith CE, Chong DL, Bartlett JD, Margolis HC (2005) Mineral acquisition rates in developing enamel on maxillary and mandibular incisors of rats and mice: implications to extracellular acid loading as apatite crystals mature. J Bone Miner Res 20: 240–249. 15647818 10.1359/JBMR.041002

[37] Yamada Y, Yamakoshi Y, Gerlach RF, Hu CC, Matsumoto K, et al (2003) Purification and characterization of enamelysin from secretory stage pig enamel. Arch Comp Biol Tooth Enam 8: 21–25.

[38] Ryu OH, Fincham AG, Hu CC, Zhang C, Qian Q, et al (1999) Characterization of recombinant pig enamelysin activity and cleavage of recombinant pig and mouse amelogenins. J Dent Res 78: 743–750. 10096449 10.1177/00220345990780030601

[39] Nagano T, Kakegawa A, Yamakoshi Y, Tsuchiya S, Hu JC, et al (2009) Mmp-20 and Klk4 cleavage site preferences for amelogenin sequences. J Dent Res 88: 823–828. 19767579 10.1177/0022034509342694 PMC2751868

[40] Krebsbach PH, Lee SK, Matsuki Y, Kozak CA, Yamada KM, et al (1996) Full-length sequence, localization, and chromosomal mapping of ameloblastin. A novel tooth-specific gene. J Biol Chem 271: 4431–4435. 8626794 10.1074/jbc.271.8.4431

[41] Fukae M, Tanabe T, Murakami C, Dohi N, Uchida T, et al (1996) Primary structure of the porcine 89-kDa enamelin. Adv Dent Res 10: 111–118. 9206327 10.1177/08959374960100020201

[42] Hu CC, Fukae M, Uchida T, Qian Q, Zhang CH, et al (1997) Cloning and characterization of porcine enamelin mRNAs. J Dent Res 76: 1720–1729. 9372788 10.1177/00220345970760110201

[43] Fukumoto S, Kiba T, Hall B, Iehara N, Nakamura T, et al (2004) Ameloblastin is a cell adhesion molecule required for maintaining the differentiation state of ameloblasts. J Cell Biol 167: 973–983. 15583034 10.1083/jcb.200409077 PMC2172447

[44] Hu JC, Hu Y, Smith CE, McKee MD, Wright JT, et al (2008) Enamel defects and ameloblast-specific expression in Enam knock-out/lacz knock-in mice. J Biol Chem 283: 10858–10871. 18252720 10.1074/jbc.M710565200 PMC2447669

[45] Yamakoshi Y, Hu JC, Fukae M, Yamakoshi F, Simmer JP (2006) How do enamelysin and kallikrein 4 process the 32-kDa enamelin? Eur J Oral Sci 114 Suppl 145–51. 16674662 10.1111/j.1600-0722.2006.00281.x

[46] Iwata T, Yamakoshi Y, Hu JC, Ishikawa I, Bartlett JD, et al (2007) Processing of ameloblastin by MMP-20. J Dent Res 86: 153–157. 17251515 10.1177/154405910708600209

[47] Chun YH, Yamakoshi Y, Yamakoshi F, Fukae M, Hu JC, et al (2010) Cleavage site specificity of MMP-20 for secretory-stage ameloblastin. J Dent Res 89: 785–790. 20400724 10.1177/0022034510366903 PMC2909333

[48] Fukae M, Yamamoto R, Karakida T, Shimoda S, Tanabe T (2007) Micelle structure of amelogenin in porcine secretory enamel. J Dent Res 86: 758–763. 17652206 10.1177/154405910708600814

[49] Simmer JP, Richardson AS, Hu YY, Smith CE, Ching-Chun Hu J (2012) A post-classical theory of enamel biomineralization... and why we need one. International Journal of Oral Science 4: 129–134. 22996272 10.1038/ijos.2012.59 PMC3464985

[50] Sharma R, Tye CE, Arun A, MacDonald D, Chatterjee A, et al (2011) Assessment of dental fluorosis in Mmp20 +/– mice. J Dent Res 90: 788–792. 21386097 10.1177/0022034511398868 PMC3092810

